# Prognostic Value of Histological Subtypes and Clinical Factors in Non-Endemic Nasopharyngeal Carcinoma: A Retrospective Cohort Study

**DOI:** 10.3390/medicina61122207

**Published:** 2025-12-13

**Authors:** Seda Sali, Candan Demiröz Abakay, Mürsel Sali, Hakan Güdücü, Fahri Güven Çakır, Birol Ocak, Ahmet Bilgehan Şahin, Alper Coşkun, Sibel Oyucu Orhan, Arife Ulaş, Adem Deligönül, Türkkan Evrensel, Erdem Çubukçu

**Affiliations:** 1 Department of Medical Oncology, University of Health Sciences, Bursa City Education and Research Hospital, Bursa 16250, Türkiye; sibeloyucu@yahoo.com (S.O.O.); drarifeulas@hotmail.com (A.U.); 2 Department of Radiation Oncology, School of Medicine, Bursa Uludağ University, Görükle, Nilüfer, Bursa 16059, Türkiye; candande@uludag.edu.tr; 3 Department of Medical Oncology, School of Medicine, Bursa Uludağ University, Görükle, Nilüfer, Bursa 16059, Türkiye; murselsali@uludag.edu.tr (M.S.); absahin@uludag.edu.tr (A.B.Ş.); ademd@uludag.edu.tr (A.D.); evrensel@uludag.edu.tr (T.E.); erdemcubukcu@uludag.edu.tr (E.Ç.); 4 Department of İnternal Medicine, Emsey Hospital, Pendik, İstanbul 34912, Türkiye; hknguducu@hotmail.com; 5 Department of İnternal Medicine, School of Medicine, Bursa Uludağ University, Görükle, Nilüfer, Bursa 16059, Türkiye; fguvencakir@hotmail.com; 6 Department of Medical Oncology, Bursa Yüksek İhtisas Training and Research Hospital, Yıldırım, Bursa 16310, Türkiye; birol08ocak@gmail.com (B.O.); alpercoskun@uludag.edu.tr (A.C.)

**Keywords:** nasopharyngeal carcinoma, histological subtype, survival, prognostic factors, non-endemic region, inflammatory indices

## Abstract

*Background and Objectives*: Nasopharyngeal carcinoma (NPC) displays marked geographic and histopathological heterogeneity, and prognostic determinants in non-endemic regions remain incompletely defined. This study aimed to evaluate the impact of clinicopathological characteristics and treatment modalities on survival outcomes among patients with stage II–IVA NPC treated with curative intent at a single tertiary cancer center. *Materials and Methods*: A retrospective analysis was conducted on 81 consecutive patients with histologically confirmed NPC treated between 2000 and 2022. Demographic, clinical, and treatment parameters were extracted from institutional records. Survival outcomes—including disease-free survival (DFS), locoregional recurrence-free survival (LRFS), distant metastasis-free survival (DMFS), cancer-specific survival (CSS), and overall survival (OS)—were estimated using the Kaplan–Meier method and compared using the log-rank test. Prognostic variables identified in univariate analysis were further assessed by multivariable Cox proportional hazards regression (Cox’s model). *Results*: The cohort included 59 men (72.8%) and 22 women (27.2%), with a median age of 50.8 years (range, 19–78). Most patients presented with locally advanced disease (T3–T4, 53.1%; N2, 60.5%; stage III–IVA, 87.7%). Non-keratinizing undifferentiated carcinoma (World Health Organization [WHO] type III) was the predominant histology (71.6%), followed by the non-keratinizing differentiated subtype (17.3%). Median DFS and OS were 94.6 and 139.4 months, respectively. According to the univariate analysis, histological subtypes and a family history of cancer were significantly associated with DFS, whereas comorbid systemic disease showed an unexpected association with longer DMFS. The multivariable Cox model identified the histological subtype as an independent predictor of disease recurrence (HR = 2.23, 95% CI: 1.00–4.94; *p* = 0.049). For OS, both histological subtype (HR = 2.40, 95% CI: 1.10–5.25; *p* = 0.029) and age at diagnosis (HR = 1.05, 95% CI: 1.02–1.09; *p* = 0.005) were independent adverse prognostic factors. *Conclusions*: In this long-term, single-center study from a non-endemic region, histological subtype emerged as the most powerful determinant of prognosis, significantly influencing both DFS and OS. Patients with non-keratinizing undifferentiated (WHO type III) carcinoma demonstrated superior outcomes compared with those with differentiated histology. Additionally, increasing age at diagnosis was independently associated with poorer OS. In contrast, inflammatory and nutritional biomarkers, the Pan-Immune–Inflammation Value (PIV) and the Prognostic Nutritional Index (PNI), showed no prognostic significance. These findings underscore the continued prognostic relevance of histopathologic classification and age and highlight the need for large-scale, standardized studies integrating Epstein–Barr virus (EBV) status and host-related factors in non-endemic NPC populations.

## 1. Introduction

Nasopharyngeal carcinoma (NPC) arises from the epithelial lining of the nasopharynx and differs from other head and neck cancers in terms of etiology, histopathology, and therapeutic approaches [1]. In 2022, an estimated 120,434 new cases and 73,482 deaths were reported globally, rendering NPC relatively uncommon compared with other cancers. The disease exhibits a striking geographic predilection, occurring predominantly in Southeast Asia—particularly Southern China—as well as in East Asia and North Africa [2]. Its etiology involves a complex interplay between genetic predisposition, Epstein–Barr virus (EBV) infection, and environmental risk factors such as tobacco exposure and consumption of salted or preserved foods [3]. According to the World Health Organization (WHO), NPC is classified into three histological subtypes: keratinizing squamous cell carcinoma (Type I), non-keratinizing differentiated carcinoma (Type II), and non-keratinizing undifferentiated carcinoma (Type III). The basaloid squamous cell subtype is also included within Type III carcinoma [4]. Type I tumors are more commonly seen in non-endemic regions, whereas those of Type III predominate in endemic areas such as Southeast Asia. Types II and III exhibit a strong association with EBV infection [4,5,6]. From a therapeutic perspective, NPC is recognized as both radiosensitive and chemosensitive. Although radiotherapy alone can achieve cure rates of approximately 90% in early-stage disease, the majority of patients—around 70%—present with locally advanced tumors at diagnosis. Even with contemporary concurrent chemoradiotherapy (CCRT) administered with curative intent, disease relapse or distant metastasis develops in nearly one-quarter of cases [7]. Chemoradiotherapy (CRT) treatment is applied as CCRT, induction chemotherapy, or adjuvant chemotherapy. Cisplatin remains the cornerstone concurrent agent with radiotherapy [8]. Recent studies demonstrate that induction chemotherapy significantly improves recurrence-free survival (RFS) and progression-free survival (PFS) compared with chemoradiotherapy alone. In the landmark phase III trial by Zhang et al., induction chemotherapy with gemcitabine plus cisplatin significantly improved 3-year recurrence-free survival compared with chemoradiotherapy alone (85.3% vs. 76.5%) [9].

## 2. Materials and Methods

**Figure 1:**
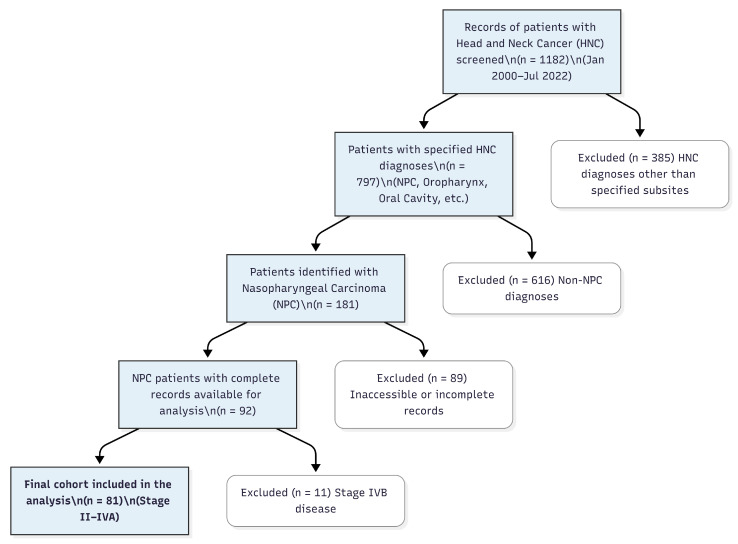
Study selection flow diagram. Among 1182 patients with head and neck cancers screened between January 2000 and July 2022, 181 cases of stage II–IVB nasopharyngeal carcinoma (NPC) were identified. Electronic medical records were accessible for 92 patients; 11 patients with stage IVB disease were excluded. Finally, 81 patients with stage II–IVA NPC who were evaluated in the medical oncology department for curative-intent chemotherapy or chemoradiotherapy were included in the final analysis.

Patients with stage II–IVA NPC who were diagnosed and received treatment at the Department of Medical Oncology, Uludağ University Faculty of Medicine, between January 2000 and July 2022, were retrospectively included in this study. A total of 1182 patients with head and neck cancer were screened during the study period. Among them, 181 patients were identified as having stage II–IVB NPC. After excluding those with inaccessible or incomplete data (*n* = 89) and those with stage IVB disease (*n* = 11), 81 patients with stage II–IVA NPC and complete clinical information were included in the final analysis (Figure 1). Eligible patients were required to be ≥18 years old, have a histologically confirmed diagnosis of nasopharyngeal carcinoma, and present with non-metastatic disease planned for curative-intent therapy. In addition, patients must have received at least one cycle of systemic chemotherapy as part of their treatment strategy. We excluded patients who were unable to receive systemic chemotherapy, or lacked sufficient and reliable electronic clinical records. Individuals who were transferred to another institution before completing treatment or follow-up were also excluded in order to maintain consistency and ensure accurate data collection within the study cohort.

Demographic, clinical, and pathological variables were retrieved from electronic health records, including age, sex, histological subtype (non-keratinizing differentiated or non-keratinizing undifferentiated carcinoma), TNM stage based on the American Joint Committee on Cancer (AJCC) 8th edition, comorbidities, lifestyle factors such as smoking and alcohol consumption, and treatment characteristics (chemotherapy, radiotherapy, and their sequencing). All included cases were restaged according to the AJCC 8th edition based on diagnostic MRI and/or PET-CT imaging performed using a clinical MRI system (Siemens Healthineers, Erlangen, Germany) and a Discovery IQ PET/CT scanner (GE Healthcare, Chicago, IL, USA). A single clinical reviewer reassessed anatomical involvement to ensure staging consistency across the study period. Histological subtype was determined from archived pathology reports following WHO classification criteria. Cases without sufficient pathological detail were recorded as unknown and excluded from subtype-specific analyses. Hematological parameters were obtained from baseline laboratory tests prior to treatment initiation. Derived inflammatory and nutritional indices were calculated as follows: Prognostic Nutritional Index (PNI) = 10 × Albumin (g/dL) + 0.005 × Lymphocyte count (/mm^3^); Pan-Immune-Inflammation Value (PIV) = (Neutrophil × Platelet × Monocyte)/Lymphocyte.

All patients had received stage-appropriate, guideline-recommended curative-intent therapies in accordance with institutional protocols and with the National Comprehensive Cancer Network (NCCN) and the European Society for Medical Oncology (ESMO) guidelines for locally advanced nasopharyngeal carcinoma. One patient had received chemotherapy alone because radiotherapy could not be tolerated.

Disease-free survival (DFS) was defined as the time from diagnosis to the first documented local, regional, or distant recurrence, or death from any cause. Locoregional recurrence-free survival (LRFS) was defined as the time from diagnosis to the first local or regional relapse. Distant metastasis-free survival (DMFS) was defined as the time from diagnosis to the detection of distant metastatic disease. Cancer-specific survival (CSS) was defined as the time from diagnosis to death attributed specifically to nasopharyngeal carcinoma. Overall survival (OS) was defined as the time from diagnosis to death from any cause. For all survival outcomes, patients without the respective event at the time of analysis were censored at death or at the data cut-off date, whichever occurred first.

### Statistical Analysis

The normality of the distribution of continuous variables was assessed using the Shapiro–Wilk test. Data showing a normal distribution were expressed as the mean ± standard deviation (SD), whereas non-normally distributed data were presented as the median (minimum–maximum). Categorical variables were summarized as frequencies and percentages. Survival analyses were performed using the Kaplan–Meier method, and survival times were reported as the mean ± standard error (SE) and the median ± SE with corresponding 95% confidence intervals (CIs). Comparisons between survival curves were made using the log-rank test. Variables found to be statistically significant in univariate analyses were subsequently included in a multivariable Cox proportional hazards regression (Cox model) to identify independent prognostic factors affecting survival outcomes. A two-tailed *p* value < 0.05 was considered statistically significant. All statistical analyses were performed using the IBM SPSS Statistics for Windows, version 25.0 (IBM Corp., Armonk, NY, USA).

## 3. Results

A total of 81 patients were included in the study, comprising 59 men (72.8%) and 22 women (27.2%). The patients’ ages ranged from 19 to 78 years, with a mean age of 50.8 ± 11.8 years. Baseline demographic, clinical, and treatment characteristics of the patients are summarized in Table 1. Non-keratinizing undifferentiated histology accounted for the majority of cases (71.6%), followed by non-keratinizing differentiated type (17.3%) and unknown variants (11.1%). Histological subtyping was available for 72 patients. Among these, no keratinizing squamous cell carcinoma (WHO Type I) cases were identified. The remaining 9 patients lacked sufficient pathological detail to determine subtype and were classified as “unknown” and excluded from subtype-specific analyses. Most patients presented with locally advanced disease: T3–T4 lesions comprised 53.1%, N2 involvement was observed in 60.5%, and stage III–IVA disease represented 87.7% of the cohort. Comorbid systemic disease was documented in 39.5% of patients. A positive family history of cancer was reported in 22.2%, while smoking and alcohol consumption were present in 42.0% and 17.3%, respectively.

Cisplatin-based chemotherapy was administered to 95.1% of the patients, and radiotherapy to 98.8%. Concurrent chemoradiotherapy (CCRT) constituted the main treatment modality (76.5%). The most frequent regimen was platinum + taxane (34.6%), followed by platinum + 5-fluorouracil (5-FU) (29.6%) and docetaxel–cisplatin–5-fluorouracil (TPF) (19.8%). Treatment sequence analysis revealed that 33.3% received CCRT followed by adjuvant chemotherapy, and 29.6% underwent induction chemotherapy followed by CCRT.

Initial symptoms in NPC are diverse and frequently non-specific, which contributes to delays in diagnosis. In our cohort, cervical swelling was the most common presenting complaint (44.4%), followed by nasal symptoms (24.7%), hearing loss (21%), visual disturbances (13.6%), and headache (9.9%). Although these symptoms reflect the anatomical pathways of tumor spread, none of them were associated with survival outcomes in our analysis (Supplementary Table S1).

**Figure 2:**
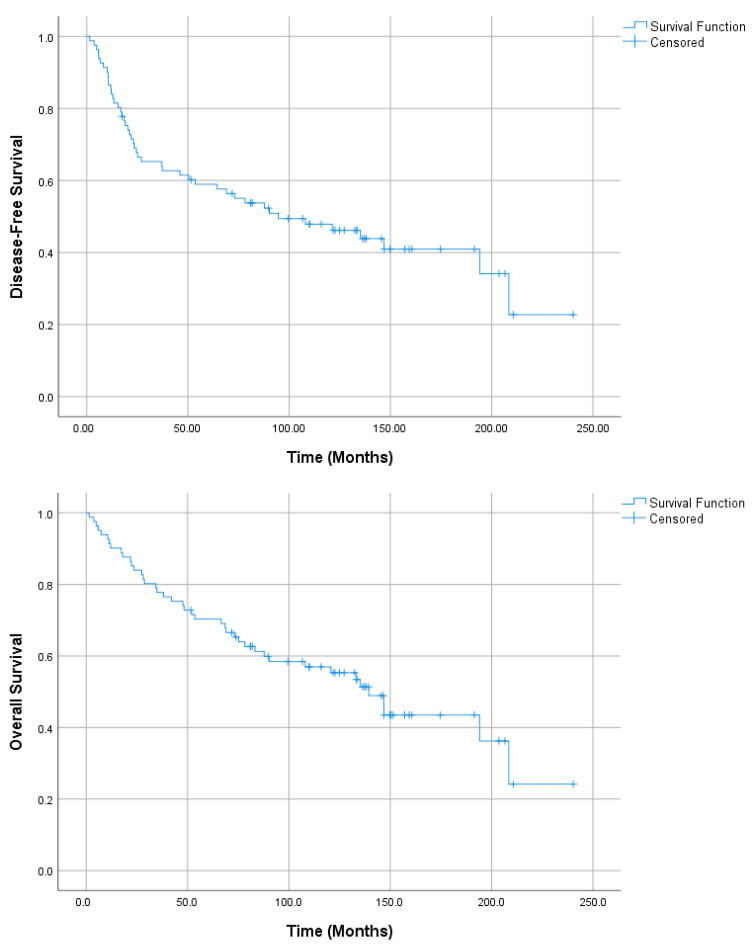
Kaplan–Meier curve for disease-free survival (DFS) and overall survival (OS) in patients with nasopharyngeal carcinoma.

Median follow-up was 99.5 months (range 1.5–240.3). Median DFS and OS were 94.6 and 139.4 months, respectively. Descriptive statistics for DFS, LRFS, DMFS, CSS, and OS are presented in Table 2 and illustrated in Figure 2.

**Figure 3:**
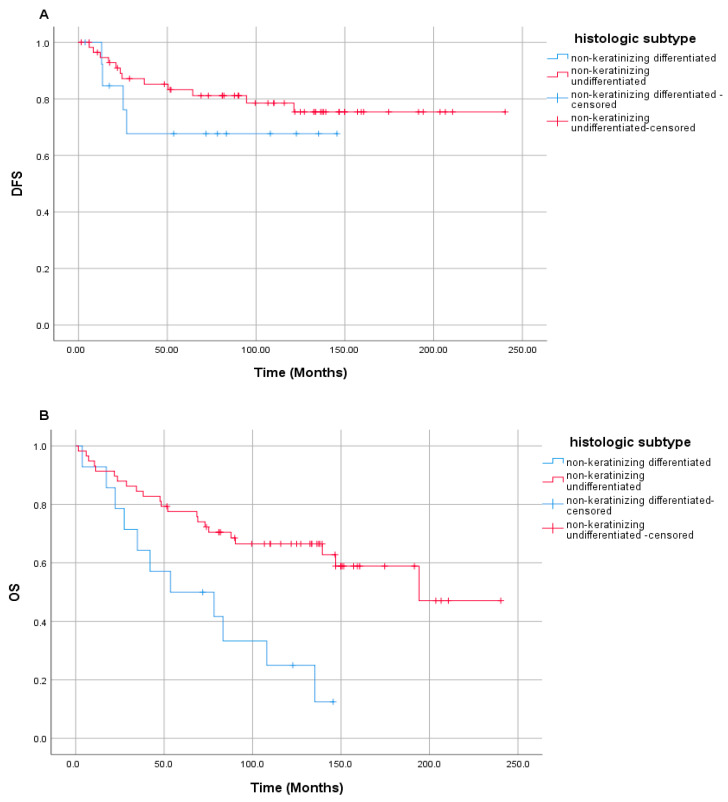
Kaplan–Meier curves for (**A**) disease-free survival (DFS) and (**B**) overall survival (OS) according to histological subtype.

In the univariate analyses, no statistically significant differences in DFS were observed with respect to sex, presenting symptoms (headache, neck mass, visual disturbance, hearing loss, nasal discharge or bleeding), T stage, N stage, TNM stage, presence of comorbid systemic disease, smoking status, alcohol use, treatment regimen, or treatment sequence. However, DFS differed significantly according to histological subtype and family history of cancer. Patients with non-keratinizing undifferentiated carcinoma had a median DFS of 194.1 ± 83.1 months, which was significantly longer than the median DFS of 27.1 ± 26.7 months observed in patients with non-keratinizing differentiated carcinoma (*p* = 0.016) (Figure 3). Similarly, patients without a family history of cancer had a median DFS of 135.2 ± 47.0 months, whereas those with a positive family history had a median DFS of 37.0 ± 14.7 months (*p* = 0.028).

When all variables were analyzed individually, no statistically significant differences were observed between groups in LRFS or CSS. Interestingly, DMFS was paradoxically longer in patients with comorbid systemic diseases compared with those without comorbidities (median DMFS, 190.1 ± 11.2 vs. 165.4 ± 15.6 months; *p* = 0.027).

Regarding OS, a significant difference was observed between histological subtypes. Patients with non-keratinizing undifferentiated carcinoma had a median OS of 194.1 months, which was significantly longer than the median OS of 53.6 ± 31.3 months observed in patients with non-keratinizing differentiated carcinoma (*p* = 0.002) (Figure 3).

In the Cox model, we included histological subtype and family history of cancer because they were significantly associated with DFS in univariate analysis. Additionally, age at diagnosis, hemoglobin level, and the inflammatory and nutritional indices (PIV and PNI) were incorporated into the multivariable model based on their clinical relevance. Among these variables, histological subtype emerged as a significant independent prognostic factor for disease recurrence. When patients with non-keratinizing undifferentiated carcinoma were used as the reference group, those with non-keratinizing differentiated carcinoma had a 2.23-fold higher risk of disease recurrence (HR = 2.23, 95% CI: 1.00–4.94; *p* = 0.049) (Table 3).

In the Kaplan–Meier analyses stratified by categorical variables, no statistically significant differences were observed in LRFS across any of the examined groups. Furthermore, a Cox model incorporating hemoglobin, age at diagnosis, and inflammatory indices (PIV and PNI) did not yield a statistically significant model (*p* = 0.394) (Supplementary Table S2).

Analysis of DMFS by categorical variables revealed a statistically significant difference only for comorbid systemic disease. Nevertheless, when comorbid systemic disease, hemoglobin, age at diagnosis, and inflammatory indices (PIV and PNI) were entered into the Cox model, the overall model failed to reach statistical significance (*p* = 0.190) (Supplementary Table S3).

Across categorical comparisons, no significant differences were detected in CSS. Similarly, the Cox model incorporating hemoglobin, age at diagnosis, and inflammatory indices (PIV and PNI) did not achieve statistical significance (*p* = 0.063) (Supplementary Table S4). However, when age at diagnosis was analyzed independently, it emerged as a significant prognostic factor; each one-year increase in age was associated with a 1.045-fold higher risk of cancer-specific mortality (*p* = 0.025) (HR = 1.05, 95% CI: 1.01–1.09) (Supplementary Table S5).

Analysis of OS across categorical variables revealed a statistically significant difference only for histological subtype (*p* = 0.002). When the histological subtype, age at diagnosis, hemoglobin, and inflammatory indices (PIV and PNI) were entered into the Cox model, both the histological subtype and age at diagnosis emerged as significant independent prognostic factors. Using non-keratinizing undifferentiated carcinoma as the reference group, patients with non-keratinizing differentiated carcinoma had a 2.40-fold higher risk of death (HR = 2.399, 95% CI: 1.10–5.25, *p* = 0.029). Additionally, each one-year increase in age at diagnosis was associated with a 1.053-fold increase in the risk of death (HR = 1.05, 95% CI: 1.02–1.09, *p* = 0.005) (Table 4).

## 4. Discussion

In our cohort of patients with stage II–IVA NPC treated with curative intent, histological subtype emerged as the most significant determinant of prognosis, demonstrating a clear impact on both DFS and OS. Patients with non-keratinizing undifferentiated (WHO type III) carcinoma had markedly longer DFS and OS compared with those with non-keratinizing differentiated (WHO type II) carcinoma. In addition, age at diagnosis was independently associated with OS, indicating that increasing age confers a higher risk of mortality even among patients receiving guideline-concordant therapy. These findings reaffirm the prognostic relevance of histopathological classification and suggest that host factors such as age continue to influence long-term outcomes despite advances in treatment strategies.

The apparently longer DMFS in patients with comorbid systemic disease is counterintuitive and most likely reflects confounding and competing-risk phenomena rather than a genuine prognostic advantage. Notably, this association did not remain significant in multivariable Cox model, and the overall DMFS model was not statistically significant, in the context of a limited number of distant failure events.

Gender distribution in NPC cohorts consistently demonstrates a marked male predominance. Studies from endemic regions typically report male-to-female ratios of approximately 70% to 30%, while similar distributions have been observed in non-endemic populations as well [10,11,12]. In our study, the male proportion was 72.8%, which aligns closely with these reports. Age patterns are also comparable across regions, with most series describing diagnosis in mid-adulthood, generally ranging from the late 40s to mid-50s [10,11,12]. In our cohort, the median age was 52 years (range: 19–78), falling within the expected range described in the literature.

Initial symptoms in nasopharyngeal carcinoma are heterogeneous and often subtle, which commonly contributes to delays in diagnosis. In our cohort, cervical swelling was the predominant presenting complaint, followed by nasal and otologic manifestations, consistent with the known anatomical patterns of tumor extension in NPC. However, despite their biological relevance, these symptoms did not appear to influence prognosis in our population (Supplementary Table S1). A recent study from a non-endemic region similarly reported cervical lymphadenopathy and nasal obstruction as the predominant symptoms at diagnosis; however, that study focused on clinical and pathological characteristics, and did not evaluate prognostic associations with survival [13]. Taken together, these findings suggest that initial symptoms primarily reflect local tumor extent rather than biological aggressiveness, emphasizing the need for heightened clinical awareness in patients presenting with subtle head and neck complaints.

The WHO classification identifies three histological subtypes of NPC. Type I (keratinizing) represents about 20% of global cases but only ~1% in endemic regions, where EBV association is rare. In contrast, Types II and III are EBV-related and account for up to 95% of cases in endemic populations [8]. The prognostic implications of histology remain debated; some studies suggest improved outcomes for WHO Type III, while others report no significant difference [10,11,14].

A recent analysis of 159 NPC patients showed that older age at diagnosis (1.03-fold increase in mortality per year) and a history of smoking were associated with worse OS. Conversely, WHO Type III histology correlated with superior OS compared with Type I. In that study, 11.7% of patients had Type I, 19.5% Type II, and 68.8% Type III tumors. Male sex predicted inferior PFS, and both advanced stage and smoking (current or former) predicted shorter RFS. Treatment modality (chemoradiotherapy alone versus induction/adjuvant therapy) did not significantly affect OS, PFS, RFS, or metastasis-free survival (MFS) [11]. Similarly, in our analysis, older age was independently associated with worse OS and, when inflammatory and nutritional variables were excluded, also with worse CSS in Cox model. Patients with WHO Type III histology had improved OS; Type I cases were absent. Most patients (71.6%) in our cohort were WHO Type III. As in previous studies, treatment modality did not influence outcomes (Supplementary Table S1).

In comparative studies of endemic versus non-endemic populations, the distribution of WHO Type II and III tumors was 12.9% and 87.1% in endemic cohorts versus 62.9% and 37.1% in Surveillance, Epidemiology, and End Results (SEER) non-endemic datasets. Smoking prevalence was higher among WHO Type II patients. Worse LRFS, DMFS, DFS, and OS were detected in patients with WHO type II in the endemic region cohort. In the other cohort, NPC-specific survival and OS were also worse in the type II arm. Multivariate analyses in this study emphasized that the histological subtype was important in predicting prognosis in endemic and non-endemic regions [12]. In our study’s multiple regression analyses, WHO type II was also associated with worse DFS and OS. Furthermore, the similar rates of type II and III in our study were 17.3% and 71.6%, respectively. The rate of smoking history was similarly higher in those with WHO type II (83.3% vs. 68.9%).

A multinational, multicenter study conducted in non-endemic regions and published in 2021 evaluated 1113 patients with NPC. Patients were stratified into an intensive-treatment cohort—comprising induction and/or adjuvant therapy combined with CRT—and a non-intensive cohort treated with radiotherapy (RT) or CRT alone. Disease-free survival (DFS) was significantly improved in non-endemic populations, particularly among those receiving intensive therapy and in patients positive for Epstein–Barr virus–encoded RNA (EBER), OS differences were not statistically significant. The analysis included patients across all disease stages, with 5-year DFS and OS rates of 65% and 84%, respectively [10]. In our cohort, only patients who received curative-intent chemoradiotherapy were included, corresponding to those with stage II–IVA disease. Depending on tumor stage, patients underwent either intensive multimodal treatment or definitive CCRT alone. In contrast to the previously mentioned multinational study, our analysis revealed no significant differences in DFS or OS according to treatment modality or sequence. The 5-year DFS and OS rates were 59% and 70%, respectively. The relatively lower survival rates observed in our series may be attributed to the exclusion of early-stage (stage I) patients.

Chemotherapy regimens in our series included platinum + taxane (34.6%), platinum + 5-FU (29.6%), TPF (triple regimen; 19.8%), and platinum + gemcitabine (4.9%); 11.1% received cisplatin monotherapy with RT. Overall, 95.1% received cisplatin, and 4.9% carboplatin. Only one patient (1.2%) did not receive RT due to poor performance status. Concurrent CRT was administered to 76.5%, while 23.5% received sequential chemotherapy and RT. Additionally, 33.3% underwent adjuvant chemotherapy after CRT, 29.6% received induction chemotherapy before CRT, and 11.1% had CRT alone (Table 1). None of these parameters—including regimen type, treatment sequence, or receipt of concurrent CRT—significantly affected DFS, LRFS, DMFS, or OS (Supplementary Table S1). Prior studies similarly found no OS advantage with more intensive regimens [10,11], although Han et al. reported a PFS benefit without OS improvement [15].

In a study investigating the effect of EBV positivity status on prognosis in NPC patients, patients were divided into two groups: EBV positive and negative. EBV negative patients were observed to have a higher rate of advanced stage disease and a higher rate of keratinizing type. Although median OS could not be reached in the study, better survival was predicted in the EBV positive group [16]. In our study, EBV testing was available in only 15 patients (12 positive, 3 negative), almost exclusively by EBER in situ hybridization performed in more recent years. The limited number of tested cases precludes subgroup comparisons for survival or stage distribution. Among WHO Type II tumors, 4/4 tested cases were EBV-positive, and among WHO Type III tumors, 8/10 were EBV-positive; however, due to small numbers and high missingness (81.5%), no clinical inference can be drawn. Therefore, EBV status was retained descriptively only, without inclusion in prognostic analyses. The limited sample prevents firm conclusions, and the literature remains inconclusive [11,16].

In a large Chinese cohort of 858 NPC patients receiving concurrent CRT, age > 45 years, advanced T and N stage, and elevated platelet-to-albumin ratio (PAR) predicted inferior OS, while histology (98.5% Type III) did not. High plasma EBV Deoxyribonucleic Acid (DNA) levels were not significant in multivariate models [17]. Another Chinese study of non-metastatic NPC identified serum albumin, systemic immune-inflammation index (SII), and monocyte count as independent prognostic factors for OS, whereas the PNI was not significant [18]. Similarly, in a study of 342 patients, elevated systemic inflammatory response index (SIRI) and metastatic disease predicted worse PFS; age ≥ 49 years and metastasis predicted inferior OS [19]. In another CRT-based study, low PIV correlated with improved OS [20]. A meta-analysis of nine studies concluded that SII and SIRI are potential prognostic biomarkers in NPC [21]. In our analysis, multivariate models including age, hemoglobin, PIV and PNI revealed no significant associations between these inflammation/nutrition markers and DFS, LRFS, DMFS, CSS, or OS (Table 3 and Table 4) (Supplementary Tables S2–S5).

The primary limitation of our study was its single-center, retrospective design. Moreover, because patients treated over an approximately 20-year period were included, EBV testing could not be consistently assessed. Since patients treated with radiotherapy alone (particularly stage T1) are typically not evaluated in the medical oncology unit, and patients with missing data or who transferred to other centers could not be included, a potential selection bias may exist. Therefore, our cohort may reflect a population with more advanced disease and complete oncologic management at our institution. Despite these constraints, the study has notable strengths, including the availability of long-term follow-up data and the ability to comprehensively evaluate presenting symptoms, treatment regimens, and therapeutic sequencing within the same cohort. Neither initiating treatment with induction chemotherapy nor administering adjuvant chemotherapy following CCRT demonstrated a significant impact on DFS, LRFS, DMFS, CSS, or OS (Supplementary Table S1).

## 5. Conclusions

This long-term analysis from a non-endemic population demonstrates that histological subtype and age remain clinically meaningful predictors of survival in nasopharyngeal carcinoma. Patients with undifferentiated non-keratinizing tumors experienced more favorable outcomes, whereas older individuals had inferior overall survival. Inflammatory and nutritional biomarkers did not show prognostic value in this cohort. These findings emphasize the importance of tumor biology and patient-related characteristics in risk assessments and highlight the need for larger, well-standardized studies to refine prognostic models and guide treatment strategies in non-endemic settings.

## Figures and Tables

**Table 1:** Baseline Demographic, Clinical, and Treatment Characteristics (*n* = 81).

Variable		Category	*n* (%)
Sex		Male	59 (72.8)
		Female	22 (27.2)
EBV status		Positive	12 (14.8)
		Negative	3 (3.7)
		Unknown/Not tested	66 (81.5)
Symptoms	Headache	Present	8 (9.9)
		Absent	66 (81.5)
		Unknown	7 (8.6)
	Neck swelling	Present	36 (44.4)
		Absent	38 (46.9)
		Unknown	7 (8.6)
	Hearing loss	Present	17 (21.0)
		Absent	57 (70.4)
		Unknown	7 (8.6)
	Nasal obstruction/discharge/bleeding	Present	20 (24.7)
		Absent	54 (66.7)
		Unknown	7 (8.6)
	Visual disturbance	Present	11 (13.6)
		Absent	63 (77.8)
		Unknown	7 (8.6)
Comorbid systemic disease		Present	32 (39.5)
		Absent	47 (58.0)
		Unknown	2 (2.5)
Smoking history		Current smoker	34 (42.0)
		Never smoked	17 (21.0)
		Former smoker	10 (12.3)
		Unknown	20 (24.7)
Family history of cancer		Yes	18 (22.2)
		No	57 (70.4)
		Unknown	6 (7.4)
Alcohol use		Yes	14 (17.3)
		No	17 (21.0)
		Former	2 (2.5)
		Unknown	47 (58.0)
T stage		T1	9 (11.1)
		T2	29 (35.8)
		T3	25 (30.9)
		T4	18 (22.2)
N stage		N0	12 (14.8)
		N1	18 (22.2)
		N2	49 (60.5)
		N3	2 (2.5)
TNM stage		Stage II	10 (12.3)
		Stage III	51 (63.0)
		Stage IVA	20 (24.7)
Histological subtype		Non-keratinizing differentiated (WHO type II)	14 (17.3)
		Non-keratinizing undifferentiated (WHO type III)	58 (71.6)
		Unknown	9 (11.1)
Cisplatin-based chemotherapy		Yes	77 (95.1)
		No	4 (4.9)
Chemotherapy regimen		RT + Platinum	9 (11.1)
		Platinum + 5-FU	24 (29.6)
		Platinum + Gemcitabine	4 (4.9)
		Platinum + Taxane	28 (34.6)
		TPF regimen	16 (19.8)
Radiotherapy received		Yes	80 (98.8)
		No	1 (1.2)
CCRT		Yes	62 (76.5)
		No	19 (23.5)
Treatment sequence pattern		CT→CCRT	24 (29.6)
		CCRT→CT	27 (33.3)
		CCRT only	9 (11.1)
		CT only	1 (1.2)
		CT→RT	13 (16.0)
		CT→RT→CT	5 (6.2)
		RT→CT	2 (2.5)

Abbreviations: CCRT = Concurrent Chemoradiotherapy; CT = Chemotherapy; EBV = Epstein–Barr Virus; RT = Radiotherapy; TNM = Tumor, Node, Metastasis; TPF = docetaxel–cisplatin–5-fluorouracil; WHO = World Health Organization; 5-FU = 5-fluorouracil.

**Table 2:** Descriptive statistics of survival outcomes.

Survival Metric	Mean ± SE (95% CI)	Median ± SE (95% CI)	Event/Total (*n/N*)
DFS	118.3 ± 11.4 (95.8–140.7)	94.6 ± 31.5 (32.9–156.2)	46/81
LRFS	193.8 ± 10.6 (172.9–214.7)	—	15/81
DMFS	186.7 ± 11.0 (165.2–208.2)	—	18/81
CSS	177.5 ± 11.1 (155.7–199.2)	—	23/81
OS	134.2 ± 10.9 (112.9–155.4)	139.4 ± 13.6 (112.8–166.0)	42/81

Abbreviations: CSS = Cancer-Specific Survival; DFS = Disease-Free Survival; DMFS = Distant Metastasis-Free Survival; LRFS = Locoregional Recurrence-Free Survival; OS = Overall Survival; SE = Standard Error. Note: ‘—’ indicates that the median survival could not be estimated.

**Table 3:** Multivariable Cox regression analysis for factors associated with disease-free survival (DFS).

Variable	*p*-Value	Hazard Ratio (HR)	95.0% CI for HR
**Age at diagnosis (years)**	0.202	1.02	0.99–1.06
**Histological subtype** Ref: non-keratinizing undifferentiated carcinoma (non-keratinizing differentiated vs. non-keratinizing undifferentiated)	**0.049**	2.23	1.00–4.94
**Family history of cancer** Ref: No (Yes vs. No)	0.193	1.86	0.73–4.71
**Hemoglobin (g/dL)**	0.566	0.93	0.72–1.19
**PIV**	0.355	1.00	0.99–1.00
**PNI**	0.671	0.99	0.93–1.05

**Model significance: *p* = 0.021**. Abbreviations: CI = Confidence İnterval; Cox model = Cox proportional hazards regression; DFS = Disease-Free Survival; HR, Hazard Ratio; PIV = Pan-İmmune-İnflammation Value; PNI = Prognostic Nutritional İndex; SE = Standard Error. Note: Bold values indicate statistical significance at *p* < 0.05.

**Table 4:** Multivariable Cox regression analysis for factors associated with overall survival (OS).

Variable	*p*-Value	Hazard Ratio (HR)	95.0% CI for HR
**Age at diagnosis (years)**	**0.** **005**	1.05	1.02–1.09
**Histological subtype** Ref: non-keratinizing undifferentiated carcinoma (non-keratinizing differentiated vs. non-keratinizing undifferentiated)	**0.029**	2.40	1.10–5.25
**Hemoglobin (g/dL)**	0.061	0. 80	0.64–1.01
**PIV**	0.497	1.00	1.00–1.00
**PNI**	0.524	1.02	0.97–1.07

**Model significance: *p* < 0.001.** Abbreviations: CI = Confidence Interval; Cox model = Cox proportional hazards regression; DFS = Disease-Free Survival; HR = Hazard Ratio; PIV = Pan-Immune-Inflammation Value; PNI = Prognostic Nutritional Index. Note: Bold values indicate statistical significance at *p* < 0.05.

## Data Availability

The clinical datasets used in this study were extracted from the institutional electronic medical record system of Bursa Uludağ University. These routinely collected clinical data were systematically analyzed to generate new research findings. Due to patient confidentiality and ethical restrictions, the raw datasets are not publicly accessible. De-identified data may be provided by the corresponding author upon reasonable request and with approval from the institutional ethics committee.

## Supplementary Materials

The following supporting information can be downloaded at https://www.mdpi.com/article/10.3390/medicina61122207/s1, Table S1. Comparison of Survival Outcomes (DFS, LRFS, DMFS, CSS, OS) According to Clinicopathological Variables. Table S2. Multivariable Cox Regression Analysis for Factors Associated with Locoregional Recurrence-Free Survival (LRFS). Table S3. Multivariable Cox Regression Analysis for Factors Associated with Distant Metastasis-Free Survival (DMFS). Table S4. Multivariable Cox Regression Analysis for Factors Associated with Cancer-Specific Survival (CSS). Table S5. Cox Regression Analysis for Factors Associated with Cancer-Specific Survival (CSS)—Age Evaluated Independently.
