# Supplementary material for: Prognostic Value of Histological Subtypes and Clinical Factors in Non-Endemic Nasopharyngeal Carcinoma: A Retrospective Cohort Study
Source: Medicina (Kaunas). 2025 Dec 13;61(12):2207. doi: 10.3390/medicina61122207 (PMC12734552; doi:10.3390/medicina61122207)
Supplement: Supplementary file 1 [file medicina-61-02207-s001.zip › medicina-4024192-supplementary.pdf]

SUPPLEMENTARY

Supplementary Table 1. Comparison of Survival Outcomes (DFS, LRFS, DMFS, CSS, OS) According to Clinicopathological Variables

Variables	DFS (p)	LRFS (p)	DMFS (p)	CSS (p)	OS (p)
Sex (Male vs Female)	0.666	0.983	0.435	0.313	0.653
Histological subtype (non-keratinizing, differentiated vs non-differentiated)	**0.016***	0.400	0.373	0.195	**0.002***
Headache (Present vs Absent)	0.387	0.173	0.412	0.514	0.638
Neck swelling (Present vs Absent)	0.625	0.720	0.682	0.074	0.325
Visual disturbance (Present vs Absent)	0.536	0.932	0.237	0.215	0.571
Hearing loss (Present vs Absent)	0.764	0.777	0.765	0.691	0.901
Nasal symptoms (Present vs Absent)	0.431	0.990	0.585	0.908	0.371
T stage (T1–T4)	0.873	0.556	0.808	0.869	0.419
N stage (N0–N2)	0.847	0.315	0.645	0.206	0.749
Family history of cancer (Yes vs No)	**0.028***	0.533	0.607	0.999	0.174
Comorbid systemic disease (Present vs Absent)	0.525	0.252	**0.027***	0.304	0.805
Smoking status (Smoker vs Non-smoker)	0.760	0.650	0.224	0.594	0.496
Alcohol use (Yes vs No)	0.724	0.792	0.567	0.705	0.886

Cisplatin use (Yes vs No)	0.565	0.782	0.942	0.821	0.307
Chemotherapy combination regimen (RT + Platinum; Platinum + 5-FU; Platinum + Gemcitabine; Platinum + Taxane; TPF regimen)	0.780	0.586	0.053	0.648	0.597
Induction/Adjuvant chemotherapy sequence	0.295	0.141	0.937	0.845	0.310
Treatment order (CT–CRT vs CRT–CT)	0.260	0.628	0.933	0.128	0.068
Concurrent chemoradiotherapy (Yes vs No)	0.661	0.828	0.568	0.373	0.459
TNM stage (II–IV)	0.386	0.184	0.970	0.771	0.197
RT initiation (Yes vs No)	0.339	0.319	0.794	0.990	0.362

*Note: p-values < 0.05 are considered statistically significant and highlighted with an asterisk (*).

***Abbreviations:** CCRT = Concurrent Chemoradiotherapy; CSS= Cancer-Specific Survival; CT = chemotherapy; DFS= Disease-Free Survival; DMFS= Distant Metastasis-Free Survival; EBV = Epstein–Barr virus; LRFS= Locoregional Recurrence-Free Survival; OS=Overall Survival; RT = Radiotherapy; TNM= Tumor, Node, Metastasis; TPF = docetaxel–cisplatin–5-fluorouracil; 5-FU=5-fluorouracil

Supplementary Table 2. Multivariable Cox Regression Analysis for Factors Associated with Locoregional Recurrence-Free Survival (LRFS)

Variable	p-value	Hazard Ratio (HR)	95% CI for HR
**Hemoglobin (g/dL)**	0.664	0.93	0.68–1.28

Variable	p-value	Hazard Ratio (HR)	95% CI for HR
Pan-immune-inflammation value (PIV)	0.364	1.00	1.00–1.00
Prognostic nutritional index (PNI)	0.312	0.95	0.87–1.05
Age at diagnosis (years)	0.403	1.02	0.97–1.08

Model significance: *p = 0.394*

---

*Abbreviations: CI: confidence interval; Cox proportional hazards regression (Cox model); LRFS: locoregional recurrence-free survival; PIV: pan-immune-inflammation value; PNI: prognostic nutritional index.

Notes: None of the variables demonstrated a statistically significant association with locoregional recurrence-free survival. HR > 1 indicates an increased risk of locoregional recurrence.

Supplementary Table 3. Multivariable Cox Regression Analysis for Factors Associated with Distant Metastasis-Free Survival (DMFS)

Variable	p-value	Hazard Ratio (HR)	95% CI for HR
Age at diagnosis (years)	0.525	1.01	0.97–1.06
Hemoglobin (g/dL)	0.145	0.79	0.58–1.08
Pan-immune-inflammation value (PIV)	0.767	1.00	1.00–1.00
Prognostic nutritional index (PNI)	0.743	1.01	0.93–1.10
Comorbid systemic disease	**0.037**	**3.90**	**1.08–14.04**

Model significance: *p = 0.190*

Abbreviations: CI: confidence interval; Cox proportional hazards regression (Cox model); DMFS: distant metastasis-free survival; PIV: pan-immune-inflammation value; PNI: prognostic nutritional index.

Notes: HR > 1 indicates an increased risk of distant metastasis.

Supplementary Table 4. Multivariable Cox Regression Analysis for Factors Associated with Cancer-Specific Survival (CSS)

Variable	p-value	Hazard Ratio (HR)	95% CI for HR
**Age at diagnosis (years)**	**0.017**	**1.05**	**1.01–1.10**
Hemoglobin (g/dL)	0.359	0.88	0.67–1.16
Pan-immune-inflammation value (PIV)	0.136	1.00	1.00–1.00
Prognostic nutritional index (PNI)	0.968	1.00	0.94–1.07

Model significance: $p = 0.063$

Abbreviations: CI: confidence interval; Cox proportional hazards regression (Cox model); CSS: cancer-specific survival; PIV: pan-immune-inflammation value; PNI: prognostic nutritional index.

Notes: HR > 1 indicates an increased risk of cancer-specific mortality.

Supplementary Table 5. Cox Regression Analysis for Factors Associated with Cancer-Specific Survival (CSS) — Age Evaluated Independently

Variable	p-value	Hazard Ratio (HR)	95% CI for HR
Age at diagnosis (years)	**0.025**	**1.05**	**1.01–1.09**

Model significance: $\boldsymbol{p = 0.020}$

Abbreviations: CI: confidence interval; Cox proportional hazards regression (Cox model); CSS: cancer-specific survival;

Notes: The variable *age at diagnosis* was evaluated independently, without inclusion of other covariates, as it demonstrated near-significant association in the multivariable model. HR > 1 indicates an increased risk of cancer-specific mortality.
